# The *Artemisia* L. Genus: A Review of Bioactive Essential Oils

**DOI:** 10.3390/molecules17032542

**Published:** 2012-03-02

**Authors:** María José Abad, Luis Miguel Bedoya, Luis Apaza, Paulina Bermejo

**Affiliations:** Department of Pharmacology, Faculty of Pharmacy, University Complutense, Ciudad Universitaria s/n, 28040, Madrid, Spain; Email: lmbedoya@farm.ucm.es (L.M.B.); luiz3apaz@gmail.com (L.A.); naber@farm.ucm.es (P.B.)

**Keywords:** *Artemisia* L., essential oil, isoprenic structure, anti-infective

## Abstract

Numerous members of the Anthemideae tribe are important as cut flowers and ornamental crops, as well as being medicinal and aromatic plants, many of which produce essential oils used in folk and modern medicine and in the cosmetics and pharmaceutical industry. Essential oils generally have a broad spectrum of bioactivity, owing to the presence of several active ingredients that work through various modes of action. Due to their mode of extraction, mostly by distillation from aromatic plants, they contain a variety of volatile molecules such as terpenes, phenol-derived aromatic and aliphatic components. The large genus *Artemisia* L., from the tribe Anthemideae, comprises important medicinal plants which are currently the subject of phytochemical attention due to their biological and chemical diversity. *Artemisia* species, widespread throughout the world, are one of the most popular plants in Chinese traditional preparations and are frequently used for the treatment of diseases such as malaria, hepatitis, cancer, inflammation and infections by fungi, bacteria and viruses. Extensive studies of the chemical components of *Artemisia* have led to the identification of many compounds as well as essentials oils. This review summarizes some of the main reports on the chemistry and anti-infective activities of *Artemisia*. Li. essential oils from the data in the recent literature (2000–2011).

## 1. Introduction

Medicinal plants are Nature’s gift to human beings to help them pursue a disease-free healthy life, and thus can play an important role in preserving health. Plants have been used as drugs by humans since thousands of years ago. Today, all the world’s cultures have an extensive knowledge of herbal medicine. Traditional medicine is based on beliefs and practices that existed before the development of so-called “modern medicine” or “scientific drug therapy”. These practices are part of a country’s cultural heritage and are transmitted orally or by written transmission.

*Artemisia* L. is a genus of small herbs and shrubs found in northern temperate regions. It belongs to the important family Compositae (Asteraceae), one of the most numerous plant groupings, which comprises about 1,000 genera and over 20,000 species. Within this family, *Artemisia* is included in the tribe Anthemideae and comprises over 500 species, which are mainly found in Asia, Europe and North America [1]. A large number of members of the Anthemideae tribe are important as cut flowers and ornamental crops, as well as medical and aromatic plants, many of which produce essential oils used in folk and modern medicine, and in the cosmetics and pharmaceutical industry [2]. Essential oils generally have a broad spectrum of bioactivity, owing to the presence of several active ingredients or secondary metabolites, which work through various modes of action. Secondary metabolism in a plant not only plays a role in its survival by producing attractants for pollinators, but it also acts as a chemical defence against predators and disease. According to the mode of extraction used, mostly distillation from the aromatic plants, essential oils contain a variety of volatile molecules such as terpenes, phenolic-derived aromatic and aliphatic components. The large genus *Artemisia* from the tribe Anthemideae comprises important medicinal plants which are currently the subject of phytochemical attention because of their biological and chemical diversity, and essential oil production.

This review, after an ethnopharmacological presentation of the genus, will focus on significant recent (2000–2011) findings on the chemistry and anti-infective activities of the essential oils from *Artemisia* species. This review has been compiled using references from major databases such as Chemical Abstract, Current Contents, Science Direct, SciFinder, Pubmed and Ethnobotanical Databases.

## 2. Ethnopharmacological Study of the *Artemisia* Genus

The 500 species of *Artemisia* are mainly found in Asia, Europe and North America. They are mostly perennial herbs dominating the vast steppe communities of Asia. Asia has the greatest concentration of species, with 150 accessions for China, 174 in the ex-USSR, about 50 reported for Japan, and 35 species of the genus found in Iran. *Artemisia* species are frequently utilized for the treatment of diseases such as malaria, hepatitis, cancer, inflammation and infections by fungi, bacteria and viruses [3]. Some *Artemisia* species are used for crafting aromatic wreaths, and as a source of essential oils used in the flavouring of vermouth; more details are given below.

Preparations of *Artemisia abrotanum* L. (“southernwood”) have been used in traditional medicine for treating a variety of disorders, including upper airway diseases. Nowadays, this perennial plant is used mainly for culinary or cosmetic purposes [4].

*Artemisia absinthium* L., commonly known as “wormwood”, is a yellow-flowering perennial plant distributed throughout various parts of Europe and Siberia, and is used for its antiparasitic effects and to treat anorexia and indigestion. The aerial parts are present in many gastric herbal preparations, in dietary supplements, and in alcoholic beverages, for example absinthe products, which are enjoying a resurgence of popularity all over the world [5].

In Afro-Asian countries, *Artemisia abyssinica* Schultz-Bip is used in folk medicine as an anthelmintic, antispasmodic, antirheumatic and antibacterial agent. This plant grows abundantly in various parts of Saudi Arabia and is locally known as “ather” [6].

*Artemisia afra* Jacq. ex Willd is a well-known medicinal plant of South Africa, where it is known as “wilde als”. It is widely used for numerous ailments including colds, coughs, diabetes, heartburn, bronchitis and asthma [7]. 

*Artemisia annua* L. (“sweet wormwood”, “qinghao”) has traditionally been used in China for the treatment of fever and chills. Though originally growing in Asia and Europe, the plant is cultivated in Africa and used as a tea for the treatment of malaria. Artemisinin has been identified as the anti-malarial principle of the plant, and artemisinin derivatives are nowadays established as anti-malarial drugs with activity towards otherwise drug-resistant *Plasmodium* infections [8]. 

*Artemisia arborescens* L. (“great mugwort”, “arborescent mugwort”) is a morphologically variable species (or mixture of species) with grey-green to silver leaves. It is native to the various habitats of the Mediterranean region, where it occurs as a shrub growing up to one metre in height. According to popular folklore, it is used as an anti-inflammatory remedy [9].

*Artemisia argyi* Levl. et Vant. is a herbaceous perennial plant with a creeping rhizome. It is native to China, Japan and the far eastern parts of the former Soviet Union. In Japan, it is known as “gaiyou” and in China as “ai ye”. It is used in herbal medicine for conditions of the liver, spleen and kidney [10]. 

The powdered leaves of *Artemisia biennis* Willd. are used as spices and in folk remedies as antiseptics. They have been applied externally in salves and washes by the native inhabitants of North American for treating sores and wounds, and internally to treat chest infections [11]. 

*Artemisia campestris* L. is a perennial faintly aromatic herb widespread in the south of Tunisia, commonly known as “tgouft”. The leaves of this plant are widely used in traditional medicine as a decoction for their antivenin, anti-inflammatory, anti-rrheumatic and antimicrobial properties [12]. 

*Artemisia cana* Pursh. is used as spice and in folk remedies as an antiseptic [13]. 

In Argentina, *Artemisia douglasiana* Besser. (“California mugwort”), which is adventitious and cultivated in the Cuyo region, is used in folk medicine and known under the common name of “matico”. The popular use of the infusion of leaves of “matico” is to treat peptic ulcers and gastrointestinal disorders [14].

*Artemisia dracunculus* L. (“tarragon”) is a perennial herb, which has a long history of use in culinary traditions. It also possesses a wide range of health benefits and has therefore been widely used as a herbal medicine. Two well-described cultivars (Russian and French) are used widely and differ in ploidy level, morphology and chemistry. The botanical and chemical constituents are closely detailed in the literature, the latter mainly focusing on its essential oil composition, which give its distinctive flavour [15]. 

*Artemisia echegaray* Hieron is commonly known in Argentina as “ajenjo”, and is used as a natural food additive [13].

Decoctions of leaves and stems of *Artemisia frigida* Willd. are used for coughs and diabetes [4]. 

*Artemisia fukudo* Makino is distributed along the shorelines of South Korea’s Jeju Island and in the south of the Korean Peninsula, Japan and Taiwan. This plant is used as a flavouring agent and in a variety of cosmetics in Korea. It also has various biological effects, including anti-inflammatory, antitumour and antibacterial properties [16].

*Artemisia haussknechtii* Boiss. is used in food as a flavouring, in perfumes and in pharmaceutical products (for its functional properties) in Iran [17].

*Artemisia herba-alba* Asso (syn. *Artemisia maritima* L., *Artemisia brevifolia* Wall.) is used in the traditional medicine of the northern Badia region of Jordan, in the form of a decoction, against fever and menstrual and nervous problems [18].

*Artemisia iwayomogi* Kitamura is a perennial herb easily found around Korea. It is called “hanin-jin” or “dowijigi” in Korean, and is traditionally used for the treatment of various liver diseases, including hepatitis [19]. 

*Artemisia judaica* L. is a perennial fragrant shrub which grows widely in the deserts and on the Sinai Peninsula in Egypt, and is a very common anthelmintic drug in most North African and Middle-Eastern countries where it is known by the Arabic name of “shih” [7]. 

The inhabitants of north-eastern of Mexico use an infusion of leaves from *Artemisia ludoviciana* Nutt. as an antidiarrheal remedy [20].

*Artemisia nilagirica* (Clarke) Pamp, commonly called “Indian wormwood”, is widely found in the hilly areas of India, where it is used as insecticide [21]. 

*Artemisia princeps* Willd. (“Japanese mugwort” or “yomogi”) is the best known *Artemisia* in Japan, where it is a fundamental ingredient of the Japanese confection “kusa-mochi”. This plant has also been used in traditional Asian medicine for the treatment of inflammation, diarrhoea and many circulatory disorders [19]. 

*Artemisia rubripes* Nakai has been used as a traditional Korean medicine for stomach ache, vomiting, diarrhoea and as a haemostatic agent [16].

*Artemisia scoparia* Waldst. & Kit. (“redstem wormwood”) is a faintly scented annual herb which is widespread and common throughout the world, particularly in southwest Asia and central Europe. The success of *A. scoparia* may be attributed to the presence of phytotoxins, the volatile essential oils, in addition to other non-volatile secondary products. It has been established that aerial parts of *A. scoparia* yield a volatile essential oil that has medicinal value. It possesses insecticidal, antibacterial, anticholesterolemic, antipyretic, antiseptic, cholagogue, diuretic, purgative and vasodilatatory activity, and is also used for the treatment of gall bladder inflammation, hepatitis and jaundice [4]. 

*Artemisia spicigera* C. Koch, known locally in Turkey as “yavsani”, is widespread in central and eastern Anatolia, and is traditionally used for skin diseases and ulcerative sores [22].

“Big sagebrush” (*Artemisia tridentata* Nutt.) is one of the most widely distributed and ecologically important shrub species in western North America. This species serves as a critical habitat and food resource for many animals and invertebrates [13]. 

*Artemisia vulgaris* L., commonly known as “mugwort”, is a perennial weed growing wild native in Asia, Europe and North America. The plant is widely used in the Philippines, where it is locally known as “herbaka”, for its antihypertensive actions. It has also been suggested to have other medicinal activities such as anti-inflammatory, antispasmodic, carminative and anthelmintic properties, and has been used in the treatment of painful menstruation (dysmenorrhoea) and in the induction of labour or miscarriage [23].

## 3. Taxonomy

The genus *Artemisia* is characterized by a wide range of morphological and phytochemical variability, which is associated with different geographical origins of the samples. The genus displays a huge ecological plasticity, with species occurring from sea level to high mountains and from arid zones to wetlands. Additionally, polyploidy is notably common and reported cytotypes differ in external morphology, anatomy, fertility and phytochemical cytogenetically [24].

## 4. Chemical Composition of Essential Oils from the *Artemisia* Genus

Essential oils are volatile, natural, complex compounds characterized by a strong odour and are formed by aromatic plants as secondary metabolites. They are usually obtained by steam or hydro-distillation, although there are several methods for extracting them. These may include the use of liquid carbon dioxide and microwaves, but mainly involve low or high pressure distillation employing boiling water or hot steam.

In nature, essential oils play an important role in the protection of plants as antibacterials, antivirals, antifungals, insecticides and also against herbivores by reducing their appetite for such plants. They also may attract some insects, thereby favouring the dispersion of pollens and seeds, or repel other undesirable insects.

Chemically, essential oils are very complex natural mixtures which can contain about 20–60 components at quite different concentrations. They are characterized by 2–3 major components at fairly high concentrations (20–70%), compared to other components present in trace amounts. Generally, these major components determine the biological properties of the essential oil. The components include two groups with different biosynthetical origins: the main group is composed of terpenes, and the other of aromatic and aliphatic constituents, all characterized by their low molecular weight.

The strong and aromatic smell of some species of *Artemisia* genus is due mainly to high concentrations of volatile terpenes, constituents of their essential oils, especially in leaves and flowers. The chemical composition of essential oils from the *Artemisia* genus has been extensively studied in several species from around the world. Many studies have shown that *Artemisia* species display significant intraspecific variations in the terpene constituents of their essential oils. In some cases, the variation in the volatile components of these plants may occur during plant ontogeny or growth at different altitudes. The quality and yield of essential oils from *Artemisia* species is influenced by the harvesting season, fertilizer and pH of soils, the choice and stage of drying conditions, the geographic location, chemotype or subspecies, choice of plant part or genotype, or extraction method. Reported constituents of *Artemisia* species are outlined in Table 1.

**Table 1:** Major essential oil components (>10%) of *Artemisia* species. ^a^ plant part: AP: aerial parts; F: flowers; FH: flower-heads; L: leaves; R: roots.

Compound	*Artemisia* species ^a^	Origin	Amount (%)	Ref.
*trans*-anethole	*A. dracunculus* (AP)	Iran	21.1	[38]
	*A. dracunculus* (AP)	Turkey	81.0	[49,50]
artemisia ketone	*A. annua* (AP)	Egypt	14.0	[28]
	*A. douglasiana* (L)	USA	26.0	[82]
	*A. pontica* (AP)	Turkey	35.6	[87]
β-bisabolol	*A. ordosica* (AP)	China	27.0	[34]
borneol	*A. abrotanum* (L)	Turkey	13.5	[87]
	*A. argyi* (F)	China	30.1	[32]
	*A. frigida* (L)	Turkey	12.3	[46]
	*A. incana* (AP)	Turkey	18.9	[80]
	*A. iwayomogi* (AP)	South Korea	18.9	[79]
	*A. nilagirica* (AP)	India	35.8	[25]
bornyl acetate	*A. argyi* (F)	China	29.8	[32]
	*A. frigida* (L)	Turkey	22.0	[46]
γ-cadinene	*A. kulbadica* (AP)	Iran	16.0	[78]
α-cadinol	*A. ordosica* (AP)	China	26.4	[34]
camphene	*A. fragans* (R)	Iran	16.9	[76]
camphor	*A. absinthium* (AP)	Ethiopia	3.7	[55]
	*A. abyssinica* (AP)	Ethiopia	31.2	[55]
	*A. afra* (L)	Ethiopia	29.1	[55]
	*A. annua* (AP)	Lithuania	42.6	[26]
	*A. annua* (L)	Ethiopia	9.6	[55]
	*A. cana* (AP)	Canada	15.9	[54]
	*A. douglasiana* (L)	USA	29.0	[82]
	*A. fragans* (R)	Iran	67.0	[76]
	*A. frigida* (L)	Turkey	40.0	[46]
	*A. frigida* (AP)	Canada	17.0	[54]
	*A. gorgonum* (AP)	Cape Verde	28.7	[47]
	*A. haussknechtii* (AP)	Iran	41.0	[39]
	*A. incana* (AP)	Turkey	19.0	[80]
	*A. iwayomogi* (AP)	South Korea	19.3	[79]
	*A. judaica* (AP)	Egypt	34.5	[45]
	*A. longifolia* (AP)	Canada	21.0	[54]
	*A. ludoviciana* (AP)	Canada	37.3	[54]
	*A. rubripes* (L)	China	26.9	[33]
	*A. santonicum* (AP)	Turkey	18.2	[49,50]
	*A. scoparia* (AP)	South Korea	11.0	[63]
	*A. sieberi* (AP)	Iran	19.5	[60,62]
	*A. spicigera* (AP)	Iran	24.6	[36]
	*A. spicigera* (AP)	Turkey	34.9	[49,50]
caryophyllene	*A. lavandulaefolia* (AP)	South Korea	16.1	[70,71]
	*A. rubripes* (L)	China	13.3	[33]
caryophyllene oxide	*A. campestris* (AP)	Lithuania	38.8	[44]
	*A. nilagirica* (AP)	India	28.6	[25]
chamazulene	*A. absinthium* (AP)	Turkey	17.8	[49,50]
	*A. arborescens* (AP)	Italy	22.7	[68]
chrysanthenone	*A. fragans* (L)	Iran	23.8	[76]
	*A. gorgonum* (AP)	Cape Verde	10.8	[47]
chrysanthenyl propionate	*A. herba-alba* (AP)	Pakistan	40.0	[43]
1,8-cineole	*A. abrotanum* (AP)	Turkey	32.6	[87]
	*A. cana* (AP)	Canada	21.5	[54]
	*A. distans* (FH)	Bulgaria	16.8	[53]
	*A. fragans* (L)	Iran	23.7	[76]
	*A. frigida* (L)	Turkey	33.8	[46]
	*A. frigida* (AP)	Canada	23.0	[54]
	*A. haussknechtii* (AP)	Iran	32.3	[39]
	*A. incana* (AP)	Turkey	14.5	[80]
	*A. iwayomogi* (AP)	South Korea	19.2	[79]
	*A. longifolia* (AP)	Canada	21.5	[54]
	*A. ludoviciana* (AP)	Canada	27.6	[54]
	*A. pontica* (AP)	Turkey	22.3	[87]
	*A. scoparia* (AP)	South Korea	21.5	[63]
	*A. spicigera* (AP)	Iran	23.3	[36]
	*A. spicigera* (AP)	Turkey	9.5	[49,50]
*p*-cymene	*A. scoparia* (L)	India	27.0	[31,65]
davanone	*A. ludoviciana* (AP)	Canada	11.5	[54]
elixene	*A. herba-alba* (AP)	Pakistan	26.0	[43]
epiglobulol	*A. ordosica* (AP)	China	25.6	[34]
eucaliptol	*A. lavandulaefolia* (AP)	South Korea	13.1	[71]
	*A. rubripes* (L)	China	15.6	[33]
	*A. sieversiana* (AP)	China	9.2	[71]
farnesene	*A. biennis* (AP)	Canada	40.0	[54]
	*A. lavandulaefolia* (AP)	South Korea	12.3	[71]
geranyl acetate	*A. aucheri* (AP)	Iran	10.7	[60]
germacrene D	*A. campestris* (AP)	Lithuania	15.0	[44]
	*A. frigida* (L)	Turkey	14.6	[46]
*cis*-lanceol	*A. ordosica* (AP)	China	25.0	[34]
limonene	*A. dracunculus* (AP)	Iran	12.4	[38]
	*A. scoparia* (L)	India	12.4	[65]
linalool	*A. annua* (AP)	India	11.9	[26]
	*A. aucheri* (AP)	Iran	44.1	[60]
methyl chavicol	*A. dracunculus* (AP)	Canada	16.2	[54]
β-myrcene	*A. absinthium* (AP)	Canada	10.8	[54]
	*A. scoparia* (L)	India	24.1	[31,65]
*trans*-ocimene	*A. biennis* (AP)	Canada	34.7	[54]
	*A. dracunculus* (AP)	Iran	20.6	[38]
9,12,15-octadecatrienal	*A. capillaris* (AP)	China	34.5	[35]
phytol	*A. capillaris* (AP)	China	33.6	[35]
α-pinene	*A. mongolica* (AP)	China	12.6	[67]
β-pinene	*A. absinthium* (AP)	Iran	23.8	[37]
	*A. scoparia* (AP)	Tajikistan	21.3	[30]
piperitone	*A. judaica* (AP)	Egypt	49.1	[45]
sabinene	*A. kulbadica* (AP)	Iran	25.1	[78]
*trans*-sabinyl acetate	*A. absinthium* (AP)	Canada	26.4	[54]
spathulenol	*A. argyi* (FH)	China	10.0	[72]
γ-terpinene	*A. scoparia* (L and R)	India	11.1	[29]
α-thujone	*A. frigida* (L)	Turkey	19.1	[46]
	*A. fukudo* (L)	Korea	48.3	[40]
	*A. pontica* (AP)	Turkey	30.1	[87]
	*A. scoparia* (AP)	Iran	81.7	[60,61]
	*A. sieberi* (AP)	Iran	10.5	[60,62]
β-thujone	*A. absinthium* (AP)	Iran	18.6	[37]
	*A. absinthium* (AP)	Canada	10.1	[54]
	*A. arborescens* (AP)	Italy	45.0	[68]
	*A. distans* (FH)	Bulgaria	9.8	[53]
	*A. frigida* (L)	Turkey	19.1	[46]
	*A. fukudo* (L)	Korea	12.7	[40]
	*A. kulbadica* (AP)	Iran	18.7	[78]
	*A. lavandulaefolia* (AP)	South Korea	13.8	[71]
	*A. scoparia* (AP)	Iran	14.5	[60,61]
	*A. sieberi* (AP)	Iran	19.8	[60,62]
	*A. spicigera* (AP)	Iran	20.7	[36]

**Figure 1:**
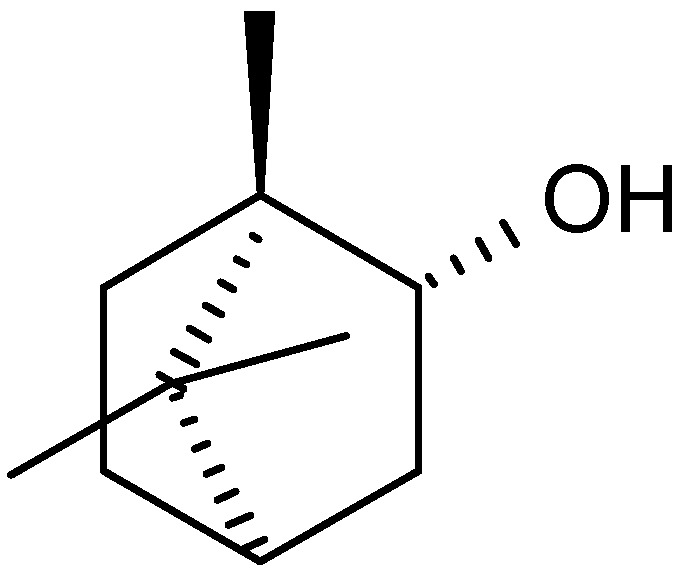
Structure of borneol.

**Figure 2:**
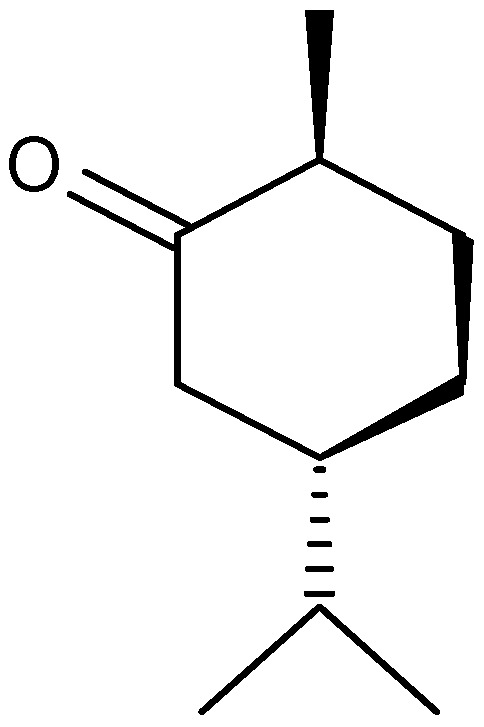
Structure of β-thujone.

**Figure 3:**
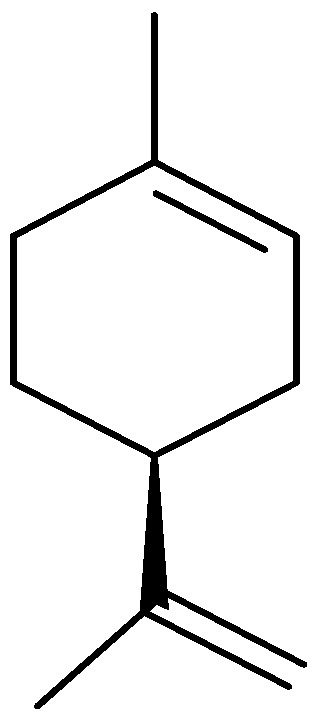
Structure of limonene.

Oils from *A. nilagirica*, a plant growing at different altitudes in Himachal Pradesh, India, were hydrodistilled and analyzed by gas chromatography (GC) and gas chromatography/mass spectrometry (GC/MS) [25]. The main constituents of the oil show variation with changes in altitude. At lower, middle and higher altitudes, the major constituents were caryophyllene oxide (28.6%), borneol (35.8%) (Figure 1) and camphor (46.9%), respectively. The characteristic compounds observed in plants from lower altitudes were 2-hexene-1-ol, β-thujone (Figure 2), thujanol, myrtenol and lynalyl acetate, while the higher altitude plants were characterized by the presence of α-pinene, β-pinene, limonene (Figure 3), linalool, γ-gurijunene, germacrane D and farnesol.

**Figure 4:**
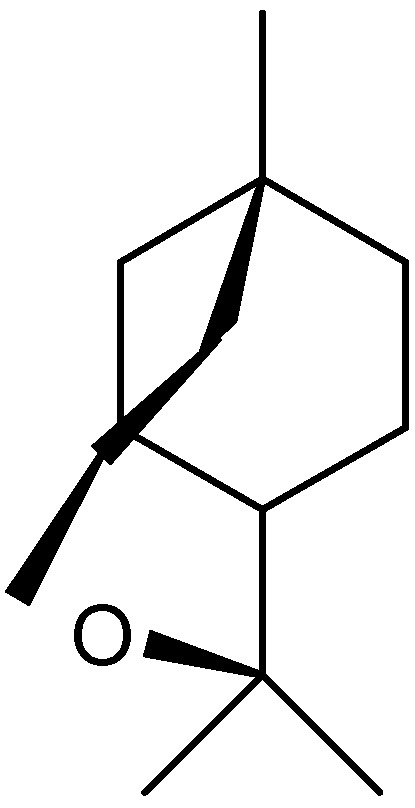
Structure of 1,8-cineole.

**Figure 5:**
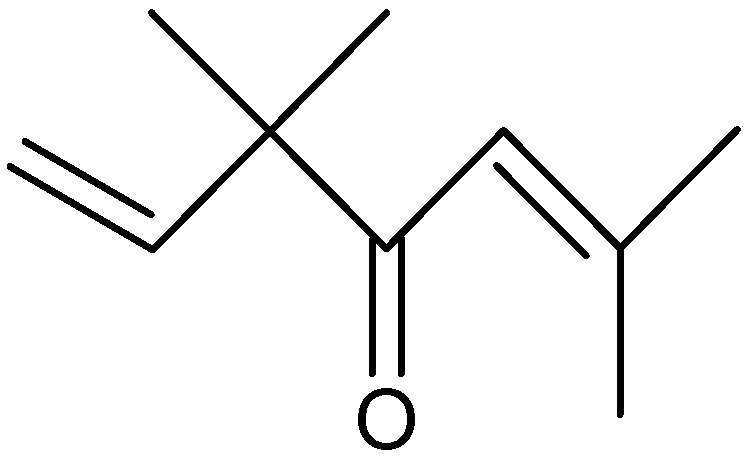
Structure of artemisia ketone.

Padalia *et al.* [26] analyzed and compared by capillary GC and GC/MS the essential oil yield and composition of the aerial parts of *A. annua* growing in Uttarakhand, India, at different stages of development. The analyses led to the identification of 81 constituents, forming 91–97.1% of the essential oil composition. However, the essential oil content was found to vary from 0.3% to 0.7% at different stages of growth. The major constituents were camphor (22.8–42.6%), 1,8-cineole (3.7–8.4%) (Figure 4), linalool (0.1–11.9%), β-caryophyllene (2–9.2%), (*E*)-β-farnese (1.3–8.5%), germacrene D (0.5–7.3%) and 1-*epi*-cubenol (0.7–5.2%). With this species cultivated in an experimental greenhouse at the Institute of Botany, Chinese Academy of Sciences, the analysis of their essential oil was also performed by comprehensive two-dimensional GC time-of-flight mass spectrometry (MS) [27]. Viuda-Martos *et al.* [28] investigated the chemical composition of this species, *A. annua*, cultivated in Egypt, and 29 components were identified, representing 93.7% of the total oil. The main constituents were 1,8-cineole (8.1%) (Figure 4) and artemisia ketone (14%) (Figure 5).

With another Indian *Artemisia* species, *A. scoparia*, Joshi *et al.* [29] investigated the essential oil composition. GC and GC/MS analysis showed the dominant presence of phenyl alkynes (61.2–85.5%), γ-terpinene (11.1%), *p*-cymene (4.5%) and (*E*)-β-ocimene (4.4%). The essential oil from the aerial parts of this species, *A. scoparia*, from Tajikistan was obtained by hydrodistillation and analyzed by GC/MS [30]. A total of 32 compounds were identified, representing 98% of the total oil composition. *A. scoparia* oil was dominated by the diacetylenes 1-phenyl-2,4-pentadiyne (34.2%) and capillene (4.9%). Other major components were β-pinene (21.3%), methyl eugenol (5.5%), α-pinene (5.4%), myrcene (5.2%), limonene (5%) (Figure 3) and β-ocimene (3.8%). The essential oil from the freshly plucked leaves of this species, *A. scoparia*, growing in wastelands around Chandigah, was extracted by hydrodistillation and analyzed by GC/MS [31]. Previously, the young and mature leaves were separated. GC/Ms analyses revealed a monoterpenoid nature (64–67%), with 44 and 31 constituents in young and mature leaves oil, respectively; β-myrcene (24.1%) and *p*-cymene (27%) were the major constituents in young and matures oil, respectively. 

Reports of the chemical composition of the essential oil from *Artemisia* species also included *A. argyi*, which is a widely used traditional Chinese medicine. Li *et al.* [32] reported for the first time the separation and identification of volatile constituents in this plant by GC/MS. The main components are borneol (Figure 1) and bornyl acetate. The chemical constituents of the essential oil from *A. rubripes* were also investigated [33]. The volatile oil was extracted from leaves by steam distillation and the components of the oil were separated and identified by GC/MS. The main components of volatile oil were camphor (26.9%), 1,8-cineole (15.6%), β-caryophyllene (13.3%) and germacrene D (5.4%). Yang *et al.* [34] investigated the chemical composition of the essential oil of another Chinese *Artemisia* species, *Artemisia ordosica* Krasch. The main composition and the composition fraction of volatile oil of this plant were analyzed by GC/MS, and 37 components, representing 90% of the total oil were identified, which mainly included 17 terpenoids, 14 alcohols, two esters, two ketones and two other components. The main constituents were β-bisabolol (27%), α-cadinol (26.4%), epiglobulol (25.6%) and *cis*-lanceol (25%). The volatiles chemical constituents of *Artemisia capillaris* Thunb., another important Chinese medicine, were also determined by GC/MS and sub-window factor analysis [35]. A total of 75 components were separated and 43 of them were qualitatively and quantitatively determined, which represented about 89% of the total content. The main constituents were 9,12,15-octadecatrienal (34.5%), phytol (33.6%) and cyclopentaneundecanoic acid (33.1%).

Members of the *Artemisia* genus have also been reported in Iran. The aerial parts of *A. spicigera* collected from Azerbaijan province, were subjected to steam distillation in Clevenger apparatus, and a light yellow residue was obtained [36]. The essential oil was submitted to careful analysis by GC and GC/MS, and the results showed the presence of approximately 17 components, with camphor (24.6%), 1,8-cineole (23.3%) (Figure 4), β-thujone (20.7%) (Figure 2) and α-thujone as the major components in this plant. The essential oil has a very strong odour and an acrid taste, and is described as neurotoxic due to its high thujone content. The composition of the volatile oil in *A. spicigera* varies widely according to the geographical location, climate, day length, soil type and cultivar. Rezaeinodehl and Khangholi [37] investigated the chemical composition of the essential oil of *A. absinthium* growing wild in Iran. The aerial parts were harvested at full bloom from an area between the villages of Deylamon and Asiabar, in Guilau province. Results showed that 28 components were identified, representing 93.3% of the oil, most of which were monoterpenes. The main components were β-pinene (23.8%) and β-thujone (18.6%) (Figure 2). The fresh aerial parts of *A. dracunculus* cultivated in Karadj (west of Tehran) were subjected to hydrodistillation using a Clevenger apparatus, and the oil analyzed by GC/MS [38]. The results revealed the presence of *trans*-anethole (21.1%), *trans*-α-ocimene (20.6%), limonene (12.4%) (Figure 3), α-pinene (5.1%), *cis*-β-ocimene (4.8%), methyl eugenol (2.2%), β-pinene (0.8%), α-terpinolene (0.5%), bornyl acetate (0.5%) and bicyclogermacrene (0.5%) as the main components. 

**Figure 6:**
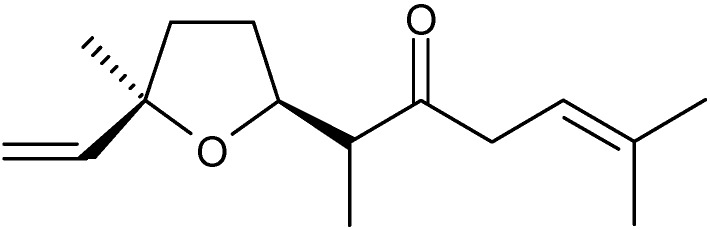
Structure of *cis*-davanone.

A novel method was developed for extraction and analysis of the volatile compounds of *A. haussknechtii* using simultaneous hydrodistillation and static head space liquid microextraction followed by GC/MS [39]. The main constituents of this essential oil included camphor (41%), 1,8-cineole (32.3%) (Figure 4), *cis*-davanone (3.7%) (Figure 6), 4-terpineol (3%), linalool (2.8%), β-fenchyl alcohol (2.7%) and borneol (2.6%) (Figure 1). Yoon *et al.* [40] investigated the chemical constituents of *A. fukudo* essential oil, a medicinal plant of the Republic of Korea, by GC/MS. The major constituents were α-thujone (48.3%), β-thujone (12.7%) (Figure 2), camphor (7%) and caryophyllene (6%).

The intraspecific chemical variability of essential oils (50 samples) isolated from the aerial parts of *A. herba-alba* growing wild in the arid zone of southeastern Tunisia was investigated [41]. Analysis by GC and GC/MS allowed the identification of 54 essential oil components. The main compounds were β-thujone (Figure 2) and α-thujone, followed by 1,8-cineole (Figure 4), camphor, chrysanthenone, *trans*-sabinyl acetate, *trans*-pinocarveol and borneol (Figure 1). Wild plants of *A. herba-alba* randomly harvested in the area of Kirchaou and transplanted by farmers for cultivation in arid zones of southern Tunisia produced an essential oil belonging to the α-thujone/β-thujone chemotype, and containing also 1,8-cineole (Figure 4), camphor and *trans*-sabinyl acetate in appreciable amounts [42]. From the essential oil from this species, *A. herba-alba* growing in Pakistan, Shah *et al.* [43] identified 25 compounds, composing 93.7% of the oil. Among these, chrysanthenyl propionate and elixene were identified for the first time from any *Artemisia* species.

**Figure 7:**
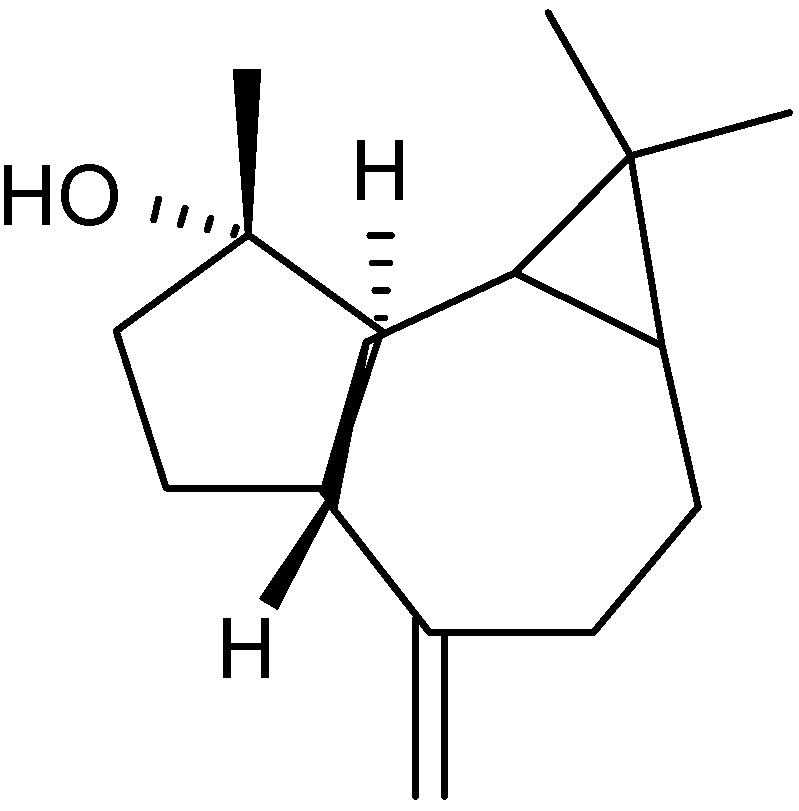
Structure of spathulenol.

Judzentiene *et al.* [44] investigated the chemical composition of the essential oil of aerial parts of *A. campestris* collected from ten different locations in Lithuania. The major component in all oils was caryophyllene oxide (8.5–38.8%), whereas compounds with the caryophyllene skeleton ranged from 10.2 to 44.5%. Other representative constituents were germacrene D (15%), humulene epoxide (8.1%), β-ylangene (7.7%), spathulenol (6.8%) (Figure 7), β-elemene (6.8%), β-caryophyllene (6.2%), junenol (6.1%) and α- or β-pinene (5.5%). Examples of other chemical constituents of the essential oils from the *Artemisia* genus also included piperitone (49.1%) and camphor (34.5%) isolated from *A. judaica* [45]. 

Members of the *Artemisia* genus have also been reported in Turkey. The composition of the essential oil from *A. frigida* in populations growing in different regions of the Republic of Kazakhstan, and the representative species *Artemisia argyrophylla* Ledeb. growing in the Altai Republic, has been investigated by GC/MS [46]. An analysis of 15 samples of the essential oil from *A. frigida* obtained over a period from 1999 to 2007 indicates that samples from different populations have similar sets of their main components: α-pinene (0.2–7.8%), camphene (1.9–5.8%), 1,8-cineole (8.9–33.8%) (Figure 4), camphor (6.7–40%), borneol (3.9–12.3%) (Figure 1), terpinen-4-ol (1.5–6.5%), bornyl acetate (1.4–22%) and germacrene D (1.4–14.6%). Some samples contain substantial amounts of α- and β-thujone (Figure 2) (in total up to 19.1%), which are completely absent in other samples. Unlike *A. frigida*, the essential oil of *A. argyrophylla* contains yomogi alcohol (1.2%), artemisia alcohol (3.1%), artemisia alcohol acetate (3.9%) and small amounts of camphor (0.2%), borneol (0.3%) (Figure 1) and bornyl acetate (0.2%). From *Artemisia gorgonum* Webb., which is used in Cape Verde folk medicine against several ailments, Ortet *et al.* [47] investigated the chemical composition of its essential oil by GC and GC/MS. A total of 111 volatile compounds, accounting for 94.9% of the essential oil were identified. The major compounds were camphor (28.7%), chrysanthenone (10.8%), lavandulyl-*Z*-methylbutanoate (9.5%), α-phellandrene (5.5%), camphene (4%) and *p*-cymene (3.4%).

## 5. Anti-infective Effects of Essential Oils from the *Artemisia* Genus

Essential oils have been largely employed for their properties which are already observed in Nature. Known for their antiseptic (bactericidal, virucidal and fungicidal) and medicinal properties and their fragrance, they are used in embalment, preservation of foods, and as microbicidal, analgesic, sedative, anti-inflammatory, spasmolytic and locally anaesthetic remedies [48]. At present, approximately 3,000 essential oils are known, 300 of which are commercially important, especially for the pharmaceutical, agricultural, food, sanitary, cosmetics and perfume industries. Essential oils or some of their components are used in perfumes and make-up products, in sanitary products, in dentistry, in agriculture, as food preservatives and additives and as natural remedies. These characteristics have remained largely unchanged to the present day, except that more is known about some of their mechanisms of action, particularly at the antimicrobial level. Lipophilic constituents of essential oils, either inhaled or topically applied, have been suggested to successfully inhibit microbial growth by means of reacting with the lipid parts of the cell membranes, rendering more permeable microbial cell membranes and mitochondria membrane, which results in death of bacterial cell after massive ion leakage and interrupting enzyme function in specific metabolic pathways. Essential oils are also able to inhibit the synthesis of DNA, RNA, proteins and polysaccharides in bacterial cells. 

A review of the literature concerning the evaluation of essential oils from the *Artemisia* genus and of the compounds isolated from them reveals that there have been many studies into their antibacterial, antifungal, antiviral and other anti-infective properties in recent years (see Table 2). These reports concern *Artemisia* species from over the world.

**Table 2:** Examples of essential oils from *Artemisia* species tested for their anti-infective capacities on standard organisms.

EO or components	Origin	Organisms	Concentrations	Ref.
*A. abrotanum*	Turkey	*Aedes aegypti*	0.22 mg	[87]
*A. absinthium*	Turkey	*Fusarium oxyosporum*	20 μg/mL	[49,50]
	Turkey	*Aspergillus niger*	600 μg/disk	[49,50]
	Serbia	*Escherichia coli*	50 μg/mL	[51]
	Serbia	*Staphylococcus aureus*	50 μg/mL	[51]
	Ethiopia	*Trypanosoma brucei*	27.9 μg/mL	[55]
*A. abyssinica*	Ethiopia	*Trypanosoma brucei*	41.8 μg/mL	[55]
	Ethiopia	*Leishmania* spp.	20 μg/mL	[56,57]
*A. afra*	Ethiopia	*Trypanosoma brucei*	77.5 μg/mL	[55]
*A. annua*	Ethiopia	*Trypanosoma brucei*	99.4 μg/mL	[55]
	India	*Tribolium castaneum*	4.1 μM/mL	[59]
*A. arborescens*	Italy	*Lysteria monocytogenes*	10^6^ CFU/mL	[68]
	Italy	*Herpes simplex virus*	2.4 μg/mL	[69]
*A. argyi*	China	*Botrytis cinerea*	1 mg/mL	[72]
*A. aucheri*	Iran	*Rhizoctonia solani*	41.4 μM/L	[60]
*A. biennis*	Canada	*Trichophyton rubrum*	10 μg/mL	[54]
	Canada	*Microsporum canis*	10 μg/mL	[54]
borneol	*A.douglasiana*	*Pseudomonas aeruginosa*	20 μg/mL	[82]
camphor	Turkish *Artemisia*	*Rhizoctonia solani*	12 mg	[49,50]
		*Sclerotium minor*	12 mg	[49,50]
		*Verticillium albo-atrum*	12 mg	[49,50]
	*A. douglasiana*	*Pseudomonas aeruginosa*	20 μg/mL	[82]
*A. cana*	Canada	*Fonsecaea pedrosol*	10 μg/mL	[54]
		*Trichophyton rubrum*	10 μg/mL	[54]
carvone	*A. herba-alba*	*Penicillium citrinum*	50 μg/mL	[85]
		*Mucora rouxii*	7 μg/mL	[85]
1,8-cineole	Turkish *Artemisia*	*Fusarium sambucinum*	20 μg/mL	[49,50]
		*Penicillium jensenii*	20 μg/mL	[49,50]
		*Verticillium albo-atrum*	20 μg/mL	[49,50]
		*Verticillium tenerum*	20 μg/mL	[49,50]
	*A. douglasiana*	*Pseudomonas aeruginosa*	20 μg/mL	[82]
	*A. annua*	*Trypanosoma brucei*	64.6 μg/mL	[75]
*A. distans*	Bulgaria	*Staphylococcus aureus*	20 μg/mL	[53]
		*Candida albicans*	20 μg/mL	[53]
*A. douglasiana*	USA	*Bacillus cereus*	0.37 μg/mL	[81]
		*Pseudomonas aeruginosa*	0.23 μg/mL	[81]
*A. dracunculus*	Turkey	*Aspergillus niger*	600 μg/disk	[49,50]
		*Fusarium acuminatum*	600 μg/disk	[49,50]
		*Acinetobacter baumanii*	600 μg/disk	[49,50]
		*Proteus vulgaris*	600 μg/disk	[49,50]
		*Pseudomonas aeruginosa*	600 μg/disk	[49,50]
*A. fragans*	Argentina	*Lysteria monocytogenes*	2.4 μg/mL	[77]
*A. frigida*	Canada	*Trichophyton rubrum*	10 μg/mL	[54]
		*Microsporum canis*	10 μg/mL	[54]
*A. lavandulaefolia*	South Korea	*Sitophilus zeamais*	11.2 mg/L	[71]
*A. longifolia*	Canada	*Microsporum canis*	10 μg/mL	[54]
		*Microsporum gypseum*	10 μg/mL	[54]
*A. ludoviciana*	Canada	*Trichophyton rubrum*	10 μg/mL	[54]
		*Microsporum canis*	10 μg/mL	[54]
*A. mongolica*	China	*Sitophilus zeamais*	7.35 mg/L	[67]
piperitone	*A. herba-alba*	*Penicillum citrinum*	2 μg/mL	[85]
		*Mucora rouxii*	1.5 μg/mL	[85]
*A. princeps*	China	*Sitophilus zeamais*	250 μg/g	[73]
	Korea	*Candida albicans*	0.5 μM/mL	[74]
*A. santonicum*	Turkey	*Alternaria alternata*	10 μg/mL	[49,50]
		*Sclerotium minor*	10 μg/mL	[49,50]
		*Brevibacillus brevis*	600 μg/disk	[49,50]
		*Acinetobacter baumanii*	600 μg/disk	[49,50]
		*Bacillus megaterium*	600 μg/disk	[49,50]
*A. scoparia*	Iran	*Callosobruchus maculates*	1.46 μg/mL	[61]
	China	*Sitophilus zeamais*	5.31 mg/L	[67]
*A. sieberi*	Iran	*Fusarium moniliforme*	750 μM/L	[60]
		*Tribolium castaneum*	16.8 μM/L	[62]
*A. sieversiana*	China	*Sitophilus zeamais*	15 mg/L	[71]
*A. spicigera*	Turkey	*Sclerotium minor*	10 μg/mL	[49,50]
		*Aspergillus niger*	600 μg/disk	[49,50]
		*Rhizoctonia solani*	600 μg/disk	[49,50]
		*Brevibacterium casei*	600 μg/disk	[49,50]
		*Micrococcus lylae*	600 μg/disk	[49,50]
α-terpineol	*A. princeps*	*Gardnerella vaginalis*	0.06 μM/L	[74]
		*Candida albicans*	0.12 μM/L	[74]
vulgarone B	*A.douglasiana*	*Botrytis cinerea*	30 μM	[83]
	*A. iwayomogi*	*Staphylococcus aureus*	10 μM	[84]

Kordali *et al.* [49,50] investigated the chemical composition, antifungal and antibacterial activities of the essential oil from four Turkish *Artemisia* species, *A. dracunculus*, *A. absinthium*, *Artemisia santonicum* L. and *A. spicigera*. The main components of these essential oils were camphor (1.4–34.9%), 1,8-cineole (1.5–9.5%) (Figure 4), chamazulene (17.8%), nuciferol propionate (5.1%), nuciferol butanoate (8.2%), caryophyllene oxide (1.7–4.3%), borneol (0.6–5.1%) (Figure 1), α-terpineol (1.6–4.1%), spathulenol (1.3–3.7%) (Figure 7), cubenol (0.1–4.2%), β-eudesmol (0.6–7.2%) and terpinen-4-ol (0.1–4.2%). The antifungal activities of these essential oils were tested against eleven plant fungi, and the results showed that all the oils have potent inhibitory effects at a very broad spectrum against all of the tested fungi. Pure camphor and 1,8-cineole (Figure 4), which are the major components of the oils, showed antifungal activity against some of the fungal species. However, compared with the antibacterial activities of all the tested oils, *A. santonicum* and *A. spicigera* oils showed antibacterial activities over a very wide spectrum. The essential oil of one of these species, *A. absinthium*, also showed antibacterial activity against common human pathogens (*Escherichia coli*, *Salmonella enteritidis*, *Pseudomonas aeruginosa*, *Klebsiella pneumoniae* and *Staphylococcus aureus* [51], and acaricidal properties [52]. 

The essential oil of *Artemisia distans* W. et K. flower heads, a plant growing in Bulgaria, was analyzed by GC and GC/MS, and assayed for its antimicrobial activity [53]. Altogether 43 components were identified in concentrations of over 0.1%, representing 93.5% of the oil composition. The main constituents were 1,8-cineole (16.8%) (Figure 4), β-thujone (9.8%) (Figure 2), sabinene (8.2%), borneol (7.5%) (Figure 1), β-pinene (6.5%) and camphor (5.8%). The oil showed moderate activity against *Staphylococcus aureus* and *Candida albicans*, and weak activity against *Salmonella typhimurium*, *Proteus vulgaris* and *Escherichia coli*. 

**Figure 8:**
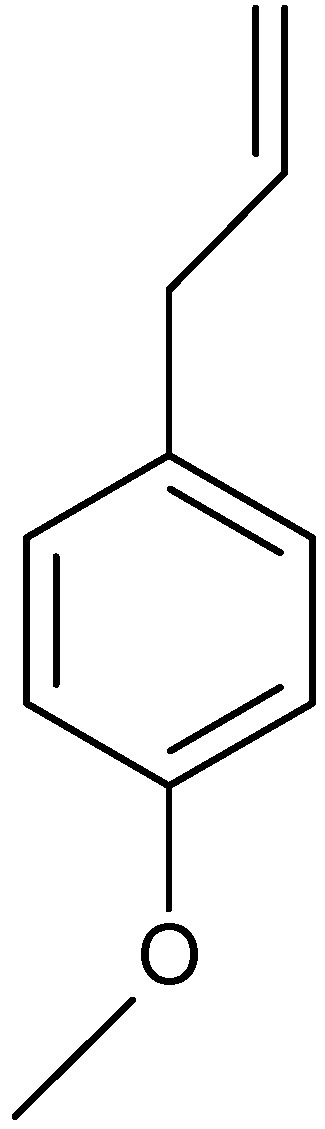
Structure of methyl chavicol.

Lopez-Lutz *et al.* [54] used GC/MS to investigate the chemical composition and antimicrobial activity of essential oil isolated from aerial parts of seven wild sages from western Canada (*A. absinthium*, *A. biennis*, *A. cana*, *A. dracunculus*, *A. frigida*, *Artemisia longifolia* Nutt. and *A. ludoviciana*). *Artemisia* oils had inhibitory effects on the growth of bacteria (*Escherichia coli*, *Staphylococcus aureus* and *Staphylococcus epidermidis*), yeasts (*Candida albicans*, *Cryptococcus neoformans*), and dermatophytes (*Trichophyton rubrum*, *Microsporum canis*, *Microsporum gypseum*, *Fonsecaea pedrosol* and *Aspergillus niger*). A total of 110 components were identified, accounting for 71–98.8% of the oil composition. High contents of 1,8-cineole (21.5–27.6%) (Figure 4) and camphor (15.9–37.3%) were found in *A. cana*, *A. frigida*, *A. longifolia* and *A. ludoviciana*. The oil of *A. ludoviciana* was also characterized by a high content of oxygenated sesquiterpenes with a 5-ethenyltetrahydro-5-methyl-2-furanyl moiety, of which davanone (Figure 6) was the main component identified (11.5%). *A. absinthium* essential oil was characterized by high amounts of myrcene (10.8%), β-thujone (10.1%) (Figure 2) and *trans*-sabinyl acetate (26.4%). *A. biennis* yielded an oil which is rich in *cis*-β-ocimene (34.7%), *trans*-β-farnesene (40%), and the acetylenes (*Z*)- and (*E*)-en-yn-dicycloethers (11%). *A. dracunculus* oil contained predominantly phenylpropanoids such as methyl chavicol (16.2%) (Figure 8) and methyleugenol.

Nibret and Wink [55] investigated the *in vitro* antitrypanosomal activity of four Ethiopian *Artemisia* species (*A. absinthium*, *A. abyssinica*, *A. afra* and *A. annua*). The essential oil from their leaves and aerial parts was tested *in vitro* against bloodstream forms of *Trypanosoma brucei brucei*. These studies validated the claims of the traditional medicinal uses of the four *Artemisia* species for the treatment of protozoal infections, in this particular case against trypanosomes. Camphor was detected in the four species and was found to be the principal compound (38.7%). The essential oil of two of these Ethiopian *Artemisia* species, *A. absinthium* and *A. abyssinica*, also showed activity against promastigote and axenic amastigote forms of two *Leishmania* strains, *Leishmania aethiopica* and *Leishmania donovani* [56,57]. These results demonstrated the potential use of both oils as a source of novel agents for the treatment of leishmaniasis. The oil of the species *A. absinthium* was also tested against eleven pathogenic bacterial strains [58]. *Lysteria monocytogenes*, *Bacillus cereus* and *Staphylococcus aureus* were the most sensitive bacteria with a minimum inhibitory concentration (MIC) of 0.14, 0.8 and 0.62 μL/mL, respectively. The essential oil of *A. annua* also presented toxic repellent and development inhibitory activities against two economically important stored product insects: *Tribolium castaneum* and *Callosobruchus maculates* [59]. Adult beetles were repelled significantly by the oil of *A. annua* at 1% concentration and in the filter paper arena test. 

Three species of the genus, *A. scoparia*, *Artemisia sieberi* Bess and *Artemisia aucheri* Boiss, widely distributed in the desert area of Iran, were screened for antifungal and anti-infective activities on some soil-borne phytopathogens and insects [60,61,62]. The essential oils were obtained by hydro-distillation of air-dried samples and their chemical composition identified by GC/MS. Oxygenated monoterpenes were the major components of the oils of the three species: α-thujone (81.7%), β-thujone (14.5%) (Figure 2) and 1,8-cineole (1.9%) (Figure 4) were the major compounds in the essential oil of *A. scoparia*; the essential oil of *A. aucheri* was rich in linalool (44.1%), geranyl acetate (10.7%), (*E*)-citral (9.7%) and (*Z*)-citral (7.7%); and the essential oil of *A. sieberi* was rich in β-thujone (19.8%) (Figure 2), α-thujone (10.5%), camphor (19.5%), verbenol (9.7%), *p*-mentha-1,5-dien-8-ol (6.4%) and davanone (5.8%) (Figure 6). Results of bioassay showed that the oils of *A. aucheri* and *A. sieberi* have stronger antifungal activity against some soil-borne pathogenic fungi such as *Rhizoctonia solani*, *Tiarospella phaseolina*, *Fusarium moniliforme* and *Fusarium solani*, while the oils of *A. scoparia* and *A. sieberi* against three coleopteran stored-product insects (*Callosobruchus maculates*, *Sitophilus oryzae* and *Tribolium castaneum*). The insecticidal constituents of these essential oils are monoterpenoids. Due to their high volatility, they have fumigant activity that might be of importance for controlling stored-product insects. 

Cha *et al.* [63] used GC/MS to analyze the chemical composition of the essential oil obtained from one of these species from South Korea, *A. scoparia*. The essential oil was rich in camphor (11%), 1,8-cineole (21.5%) (Figure 4) and β-caryophyllene (6.8%). The essential oil and its major compounds were tested for their antimicrobial activity against fifteen different genera of oral bacteria, exhibiting various degrees of growth inhibition. The essential oil from this species *A. scoparia* from India also presents phytotoxic effects against five weed species, suggesting its possible use as a bioherbicide [64]. GC/MS analysis revealed the essential oil (yield 0.84%) is a complex mixture containing 19 monoterpenes, seven sesquiterpenes and 15 other compounds (aliphatic alcohols, ketones, aromatic hydrocarbons and esters) [65]. The main monoterpenes were β-myrcene (30.2%), *p*-cymene (12.8%) and limonene (12.4%) (Figure 3). The three monoterpenes exhibited phytotoxicity and reduced germination, seedling growth and chlorophyll content of *Arena sativa* and *Triticum aestivum* in a dose-dependent manner. The essential oil of *A. scoparia* and its active constituents inhibits plant growth by generating reactive oxygen species (lipid peroxidation, membrane integrity and amounts of conjugated dienes and hydrogen peroxide), and causing oxidative damage [66]. Inhibition of germination and root growth by oil from foliage of *A. scoparia* suggests that under natural conditions, these volatile terpenes may emanate from the plant, enter the soil, and may be involved in suppression of associated vegetation, thus resulting in the formation of its monospecific strands. The essential oil from this species, *A. scoparia*, together with *Artemisia mongolica* (Fisch. ex Besser) Nakai, was also found to possess insecticidal activity against the maize weevil *Sitophilus zeamais* [67]. The main constituents of *A. mongolica* essential oil were α-pinene (12.6%), germacrene D (8.3%) and γ-terpinene (8.1%).

Militello *et al.* [68] investigated the chemical composition of essential oil from *A. arborescens* growing wild in Sicily, Italy. Essential oil extracted by steam distillation was also examined for its capability to inhibit some food-borne pathogen bacteria. A total of 43 compounds, accounting for 93.7% of the total oil, were identified by GC and GC/MS. Oxygenated monoterpenes (57.3%) constituted the main fraction, with β-thujone (45%) (Figure 2) as the main compounds, followed by the sesquiterpene hydrocarbon chamazulene (22.7%). Undiluted essential oil showed a large inhibition spectrum against strains of *Lysteria monocytogenes*, whilst it was ineffective against enterobacteria and *Salmonella*; thus, it may represent a natural preservative, alternative to the common chemical additives. This essential oil also present antiherpes virus activity, principally due to direct virucidal effect [69]. The mode of action of the essential oil as an antiherpes virus agent appears particularly interesting due to its ability to inactivate the virus and to inhibit cell-to-cell virus diffusion. 

The chemical components of the essential oil obtained from *Artemisia lavandulaefolia* DC. from South Korea were analyzed by GC/MS, and 99 compounds, accounting for 94.9% of the essential oil, were identified [70]. The major compounds were β-caryophyllene (16.1%), *cis*-chrysanthenol (7%), 1,8-cineole (5.6%) (Figure 4), borneol (5.3%) (Figure 1), *trans*-β-farnesene (5.1%), camphor (4.9%), yomogi alcohol (4.5%), α-terpineol (3.9%) and α-humulene oxide (3.3%). The essential oil and some of its main compounds were tested for antimicrobial activity against fifteen different genera of oral bacteria. The essential oil exhibited considerable inhibitory activity effects against all obligate anaerobic bacteria tested (MIC values of 0.025 to 0.05 mg/mL), while its major compounds demonstrated different degrees of growth inhibition. This plant, together with *Artemisia sieversiana* L. from China, was also found to possess insecticidal activity against the maize weevil *Sitophilus zeamais* [71]. The essential oil of the aerial parts of the two plants was obtained by hydro-distillation and analyzed by GC and GC/MS. The main components of *A. lavandulaefolia* were β-caryophyllene (15.5%), β-thujone (13.8%) (Figure 2), 1,8-cineole (13.1%) and *trans*-β-farnesene (12.3%), and the principal compounds identified in *A. sieversiana* oil were 1,8-cineole (9.2%), geranyl butyrate (9.2%), borneol (7.9%) (Figure 1) and camphor (7.9%).

Reports on the chemical composition and anti-infective activity of other *Artemisia* species from China were also found in the literature. The essential oil of *A. argyi* obtained by supercritical carbon dioxide extraction and hydro-distillation was analyzed by GC/MS to characterize its components, and also tested for antifungal activity [72]. A total of 61 compounds were identified, of which the most abundant were 1,8-cineole (4.4%) (Figure 4), borneol (3.5%) (Figure 1), terpinol (10.1%), spathulenol (10%) (Figure 7), caryophyllene oxide (6.5%), juniper camphor (8.7%), chamazulene (2%) and camphor (3.4%). The essential oil extracted by these two methods exhibited antifungal activity against *Botrytis cinerea* and *Alternaria alternata*, two common storage pathogens of fruits and vegetables. The inhibition of these fungi was 93.3% and 84.7% for oil extracted by hydrodistillation when exposed to a concentration of 1 mg/mL, while values of 70.8% and 60.5% were observed from oil extracted by supercritical carbon dioxide. The essential oil from *A. princeps*, a Chinese medicinal plant widespread in China, exhibited repellent activities against the beetles *Sitophillus orizae* and *Bruchus rugimanus* at concentrations ranging from 250 to 1000 μg/g [73]. The essential oil from this species, *A. princeps*, and its main constituents, 1,8-cineole and *α*-terpineol, also ameliorated bacterial vaginosis and vulvovaginal candidiasis in mice by inhibiting bacterial growth and nuclear factor-κB activation [74]. From the two compounds, α-terpineol most potently inhibited the growths of *Gardnerella vaginalis* and *Candida albicans* with MIC values of 0.06 and 0.125% (v/v), respectively. From another Chinese *Artemisia* species, *A. annua*, 1,8-cineole (Figure 4) has been identified as an anti-infective constituent [75]. The compound inhibited the growth of *Trypanosoma brucei brucei*, the causative agent of Nagana epidemic, with an inhibitory concentration 50 (IC_50_) value of 64.6 μg/mL.

**Figure 9:**
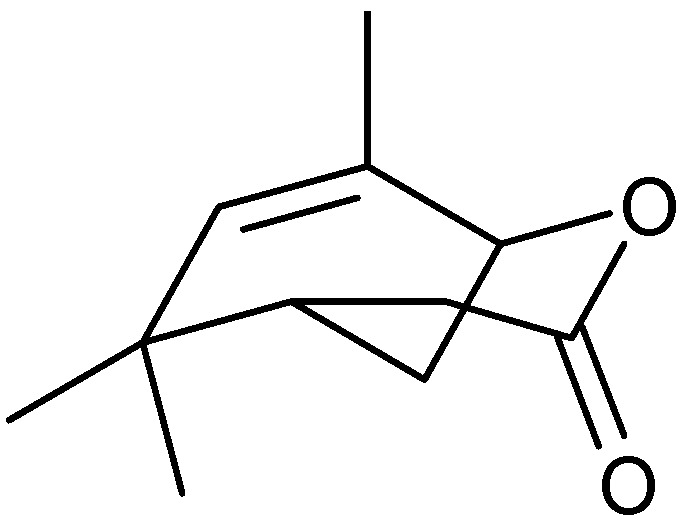
Structure of filifolide A.

Shafaghat *et al.* [76] investigated the composition and antibacterial activity of the essential oil from *Artemisia fragans* Willd from Iran. Hydro-distillation of leaves and roots of this species yielded 0.9% and 0.1% essential oils, respectively. GC/MS analysis allowed identification of 19 components, comprising 91.1% of the total oil from the leaves, while only nine compounds (93.8%) were identified in the roots. The main components of the leaf were chrysanthenone (23.8%), 1,8-cineole (23.7%) (Figure 4), β-caryophyllene (9.6%), *p*-cymene (7.7%), filifolide A (5.7%) (Figure 9) and filifolone (5–7%). In the root oil, the main constituents were camphor (67%) and camphene (16.9%). Both essential oils showed antibacterial activity against two Gram-positive and one Gram-negative bacteria. The essential oil of this species, *A. fragans*, collected from Argentina, was also active against bacterial species of significant importance in food hygiene [77]. The hydro-distilled volatile oil from another *Artemisia* species from Iran, *Artemisia kulbadica* Boiss & Buhse, also showed antimicrobial activity against six bacterial and one fungal strain [78]. Using GC/MS, 27 compounds were identified, representing 92.9% of the total oil. Sabinene (25.1%), β-thujone (18.7%) (Figure 2) and γ-cadinene (16%) were the main components.

The chemical composition of the essential oil from *A. iwayomogi* from South Korea was analyzed by means of GC and GC/MS, and 85 constituents were identified, representing 96.2% of the total oil [79]. Camphor (19.3%), 1,8-cineole (19.2%) (Figure 4), borneol (18.9%) (Figure 1), camphene (4.6%) and β-caryophyllene (3.4%) were found to be the major components. The oil exhibited antibacterial activity against six Gram-positive and six Gram-negative bacteria in tests using the broth dilution methods.

The essential oil of *Artemisia incana* (L.) Druce from Turkey was also tested for its antimicrobial activity against 44 different food-borne microorganisms, including 26 bacteria, 15 fungi and three yeast species [80]. The oil, obtained by hydrodistillation, exhibited considerable inhibitory effects against all species tested. GC and GC/MS was used to characterize 63 compounds representing 97.2% of the total components. Camphor (19%), borneol (18.9%) (Figure 1), 1,8-cineole (14.5%) (Figure 4), bornyl acetate (7.8%), camphene (4.9%) and α-thujone (4.8%) were identified as the predominant components.

The antifungal, antibacterial and insect repellent activities of the essential oil from *Artemisia mendozana* DC., a medicinal plant from the central Andes in Argentina, were also investigated [81]. However, the oil showed only moderate activity. 

**Figure 10:**
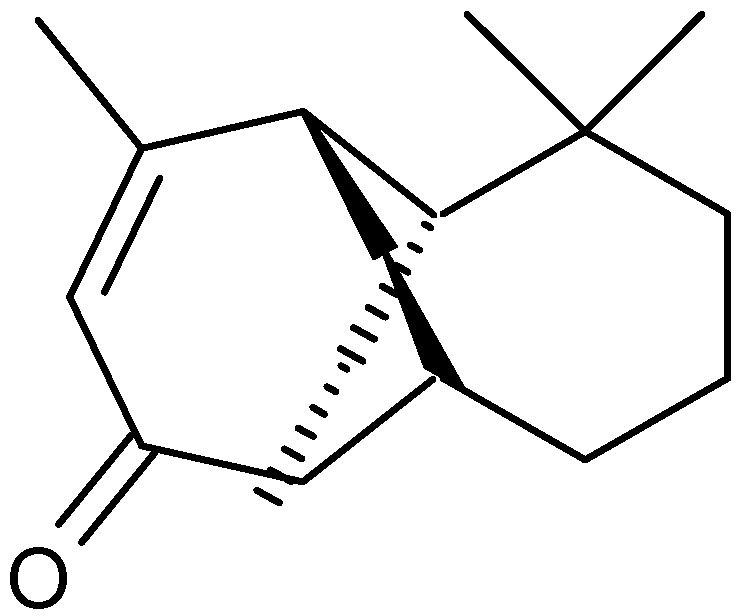
Structure of vulgarone B.

The leaf oil of *A. douglasiana* has been shown to be an efficacious complementary herbal treatment for chronic bladder infection. The oil has been analyzed by GC/MS and the major components found to be camphor (29%), artemisia ketone (26%) (Figure 5), artemisia alcohol (13%), α-thujone (10%), 1,8-cineole (8%) (Figure 4) and hexanal (5%) [82]. The leaf oil and the major components have been tested for antimicrobial activity against *Bacillus cereus*, *Staphylococcus aureus*, *Escherichia coli*, *Pseudomonas aeruginosa*, *Candida albicans* and *Aspergillus niger*. The essential oil shows limited antimicrobial activity *in vitro*, so it is unclear if the oil exerts a direct antimicrobial effect*in vivo* or plays some role in stimulating the host defences. From this species, vulgarone B (Figure 10) has been identified as the active antifungal constituent [83]. One of the possible modes of this antifungal compound may be due to their role as Michael-type acceptor for biological nucleophiles. For the studies of structure-activity relationships, it was postulated that the α,β-unsaturated carbonyl group is essential for the antifungal activity of vulgarone B. This compound, vulgarone B, has also been reported in the essential oil from *A. iwayomogi* as an antibacterial constituent [84]. The antibacterial activities of vulgarone B have been demonstrated against some antibiotic-susceptible and -resistant human pathogens, and the antibiotic mechanism involved might be related to DNA cleavage. 

The antifungal activity of another *Artemisia* species, *A. herba-alba*, was found to be associated with two major volatile compounds isolated from the fresh leaves of the plant [85]. Carvone and piperitone were isolated and identified by GC/MS. Antifungal activity was measured against *Penicillium citrinum* and *Mucora rouxii*. The antifungal activity of the purified compounds (carvone and piperitone) was estimated to be effective against *Penicillium citrinum* (IC_50_ of 5 and 2 μg/mL), and against *Mucora rouxii* (IC_50_ of 7 and 1.5 μg/mL), respectively. The essential oil from this species, *A. herba-alba*, growing wild in southwest of Tunisia, also presents antimicrobial activity against six bacterial strains and three fungal strains, with IC_50_ values of 8–51 μg/mL [86].

Examples of other *Artemisia* species with anti-infective properties also included *A. abrotanum* and *Artemisia pontica* L., which present repellent activity against *Aedes aegypti* [87]. The oil was obtained by hydro-distillation and analyzed by GC and GC/MS. The main constituents of *Artemisia* oil were as follows: in *A. abrotanum* 1,8-cineole (32.6%) (Figure 4), borneol (13.5%) (Figure 1), presil-phiperfolan-9α-ol (10.2%) and *p*-cymene (8%); and in *A. pontica* artemisia ketone (35.6%) (Figure 5), α-thujone (30.1%), 1,8-cineole (22.3%) (Figure 4) and β-thujone (3.7%) (Figure 2).

## 6. Conclusions

This review summarizes and characterizes the importance of essential oils found from a wide range of *Artemisia* species. A number of compounds of these oils (and the oils themselves) have medicinal and (ethno-) pharmacological properties. The potential for developement of leads from *Artemisia* continues to grow, particularly in the area of infectious conditions. The information summarize here is intended to serve as a reference tool to people in all fields of ethnopharmacology and natural products chemistry.
